# Association of biomass fuel smoke with respiratory symptoms among children under 5 years of age in urban areas: results from Bangladesh Urban Health Survey, 2013

**DOI:** 10.1186/s12199-019-0827-3

**Published:** 2019-11-27

**Authors:** Md. Hasan, Sadia Tasfina, S. M. Raysul Haque, K. M. Saif-Ur-Rahman, Md. Khalequzzaman, Wasimul Bari, Syed Shariful Islam

**Affiliations:** 1 Department of Public Health and Informatics, Bangabandhu Sheikh Mujib Medical University, Dhaka, Bangladesh; 2 School of Public Health, Independent University, Dhaka, Bangladesh; 3 Health Systems and Population Studies Division, icddr,b, Dhaka, Bangladesh; 4 Department of Statistics, University of Dhaka, Dhaka, Bangladesh; 5 Department of Public Health and Health Systems, University of Nagoya, Nagoya, Japan

**Keywords:** Biomass fuel smokes, Respiratory symptoms, Under-five children, Urban health, Bangladesh

## Abstract

**Background:**

Little is known regarding the effect of exposure to biomass fuel smoke inhalation on respiratory symptoms in the Bangladeshi population which is a major health hazard in most of the developing countries. This study aims to explore the association between respiratory symptoms and biomass fuel smoke exposure among children under 5 years of age.

**Methods:**

Data were extracted from the Bangladesh Urban Health Survey conducted in 2013. A total of 10,575 mothers with at least one surviving children were selected. Respiratory symptoms among children under 5 years of age were considered as the primary outcome. Sequential multiple logistic regression models were used to observe the association between respiratory symptoms and biomass fuel smoke exposure adjusting the effect of residential factors and mother and child characteristics.

**Results:**

Around 40% of the mothers exclusively used biomass fuel irrespective of the kitchen location and 54% of them were habituated in indoor cooking. The prevalence of respiratory symptoms of under-five children among in-house and outdoor biomass fuel users was 23.0% and 21.9%, respectively. Results of fitted multiple logistic regression models showed that the odds of having respiratory symptoms among children under 5 years of age were increased due to in-house biomass fuel use [OR = 1.18; 95% CI, 1.04–1.36] compared with the non-biomass user. An increased risk of respiratory symptoms was also significantly associated with mother’s birth complication [OR = 1.51; 95% CI, 1.36–1.67], non-government organization (NGO) membership of mothers [OR = 1.32; 95% CI, 1.16–1.51], age of the child (6–23m) [OR = 1.29; 95% CI, 1.10–1.52], and nutritional status (stunting) [OR = 1.18; 95% CI, 1.06–1.31].

**Conclusion:**

This study found the use of in-house biomass fuel as a significant risk factor associated with respiratory symptoms of children under 5 years of age. More longitudinal studies should be designed to establish a causal relationship between HAP (household air pollution) and respiratory symptoms among children with more direct measures of HAP and clinical procedure.

## Introduction

Thermal energy is an essential item for cooking, lighting, and heating, etc. which are the basic needs of human beings. One of the main sources of this energy in developing countries is biomass fuel (i.e., wood, charcoal, animal dung, and agriculture residues) where people can hardly afford the cost of other types of expensive fuel (i.e., liquefied petroleum gas (LPG), electricity, biogas, ethanol gel, plant oils) [1]. Globally, 52% of the population counts on biomass fuel as the main source of energy for their daily cooking [2]. In developing countries, household use of biomass fuel covers almost 7% of primary energy demand [2] and approximately 3 billion people depend on solid fuel (biomass and coal) for their daily household work which is expected to increase until by 2030 [3]. Such use of biomass fuel for cooking and heating leads to incomplete combustion which is the main source of household air pollution (HAP). HAP is considered as a prime health concern as it has already crossed the standard tolerable limit [3]. Each year, there are approximately 3.8 million premature deaths from illness caused by HAP due to the inefficient use of solid fuels [4].

Women and children are at higher risk of exposure to the polluted air because of more household involvement than their counterparts [5]. According to the World Health Organization (WHO), 2.6% of global health hazard is attributable to HAP from solid fuel which is preventable [5]. In low and middle-income countries (LMIC’s), HAP is one of the leading causes of death from non-communicable diseases (NCDs) [4]. Among children under 5 years of age, almost 19% of deaths occur due to acute respiratory infection (ARI) and it is the second common cause of death in this age group [6]. Recent studies revealed that the use of solid fuels was significantly associated with neonatal mortality [7], infant mortality [7], under-five child mortality [8] and low birth weight (LBW) [7]. In Bangladesh, almost 73% of households use biomass fuel as the main power source of cooking [9]. On the contrary, about one-fourth of the deaths among children under 5 years of age are associated with ARI, among which almost 43% are postneonatal (29 days–11 months) deaths [10].

Bangladesh is a developing country that is undergoing rapid urbanization [11]. The highest peak of the rural population was 106 million in 2016 which is now declining. In 2015, the urban population was 54 million which will be 81.4 million in 2029 [11] and Bangladesh will be an urban country by 2039 [12]. The urban population is so diverse and varied in terms of living and economic conditions and is characterized by large inequalities in health-related conditions.

Among the households in the urban area in Bangladesh, around 65% of people use biomass fuel as their main cooking fuel ingredients and almost 60% of households cook inside their house [12]. A study conducted on HAP revealed that a high concentration of air pollutants emitted from biomass fuel used in the kitchen [13]. In the winter season, the average concentration of carbon mono oxide (CO), carbon dioxide (CO_2_), dust particles, and NO_2_ during cooking was 7.6 ppm, 662 ppm, 1.051(mg/m^3^), and 80.2 (μg/m^3^) respectively [13]. So HAP is becoming an important public health concern in this country. The existing literature has got very little information on ARI of children under 5 years of age who are exposed to biomass fuel smoke in urban Bangladesh, differentiating urban areas as city corporation slum and non-slum; and other urban areas. In general, the WHO recommended that the definition of ARI [14] is used to detect ARI cases. But in developing countries like Bangladesh, where clinical data on ARI are usually not available or very weak, the symptomatic definition of illness was widely used to provide a fairly good way of assessing symptoms of ARI in several population-based studies [12, 15, 16]. In this study, an attempt has been made to examine the association between the use of biomass fuel in daily cooking and the presence of respiratory symptoms of children under 5 years of age.

## Methods and materials

### Study area

This study was conducted in the urban areas of Bangladesh, which is divided into three groups: City Corporation slum, City Corporation non-slum, and other urban areas (district municipalities and large towns/Paurashavas with population over 45,000 habitants) [12].

### Study population and design

In this cross-sectional study, data have been extracted from the Urban Health Survey (UHS) 2013 where a three-stage cluster sampling technique was used. The detailed description of the survey methodology was published in the report [12]. In this survey, 450 Mohallas were randomly selected from City Corporation and another 184 from other urban areas. “Mahalla” is the lowest administrative division in the cities in Bangladesh [12]. A total of 53,790 households were selected for this survey from where 49,228 women were successfully interviewed. For this study, a total of 10,575 women age 15–49 years having at least one alive child under 5 years of age were selected for analysis. Multiple births of a mother were reshaped statistically so that a single observation was obtained for each multiple births. In analyzing data, it was assumed that observations are independent.

### Data management

#### Outcome variable

The main variable of interest in this study was respiratory symptoms. Respiratory symptoms were defined as whether the children under 5 years of age had been suffering from cough during the last 2 weeks preceding the survey, shortness of breathing or rapid breathing, a problem in the chest including blocked, or running nose. Children who experienced at least one of the above-mentioned criteria were considered as having respiratory symptoms.

#### Exposure variable

The main exposure variable was the type of fuel used along with the place of cooking (non-biomass user, in-house biomass user, and outdoor/separate house biomass user). Non-biomass fuel or material included electricity, liquefied petroleum gas, natural gas, and biogas; and biomass fuel included coal, lignite, charcoal, wood, straw/shrubs/grass, agricultural crop, and animal dung [17].

#### Other key variables

Besides these exposures of biomass fuel uses, a range of potential confounders were selected based on previous studies demonstrating a significant impact on respiratory symptoms, which are participant’s residential factors [13, 18], mother’s characteristics [7, 19], and child characteristics [18, 19]. Participants residential factors included area of the residence (city corporation: slum; city corporation: non-slum; and other urban areas) [12], migration status (migrated and non-migrated), toilet facility (improved and non-improved) [12], source of water (piped water, tube well, and others), floor materials (concrete/cement and other materials), garbage disposal (improved and non-improved) [20]; mother’s characteristics included mother’s education (no formal education, primary, secondary, and above), age (less than 19, 20–29, 30, and above), birth complication of mothers, current employment status, number of children, exposure to media, Non-Government Organization (NGO) membership and place of handwashing (observed and non-observed) status [12]. Definitions of these variables were given in Additional file 1. The birth complication was defined if the mother experienced any of the following complications during delivery or after delivery. The complications are severe headache/blurred vision; convulsion/fits; high BP; severe bleeding; leaking membrane; mal-presentation; prolonged labour; retained placenta; high fever with smelly discharge, and edema [12]. NGO membership means if the mother belongs to any of the given organization which provides financial support with interest such as Grameen Bank, Bangladesh Rural Advancement Committee (BRAC), Bangladesh Rural Development Board (BRDB), Association for Social Advancement (ASA), and Proshika [12]. Child characteristics include the age of the child in months (0–5, 6–23, 24–59), birth order (1, 2, and 3+), gender, and nutritional [21] status (stunting) of the child. Details of these factors were given in the Bangladesh Urban Health Survey [12] and core questions on drinking water and sanitation for the household survey conducted by WHO and United Nations International Children’s Emergency Fund (UNICEF) [20].

### Data analysis

Descriptive statistics of outcome variables and exposure variables were presented in frequency and percentages. Participant’s residential factors and characteristics of the mother and child were presented in tabular form with row percentage distribution and chi-square test was performed to examine the association between respiratory symptoms and each explanatory variable. Sequential multiple logistic regression modelling had been used to examine how the influence of biomass fuel on respiratory symptoms changes with the adjustment of different sets of covariates [22]. For this purpose, four logistic regression models had been considered, which were Model 1: Only use of biomass fuel; Model 2: Model 1 + Residential factors; Model 3: Model 2 + Mother’s characteristics; and Model 4: Model 3 + Child’s characteristic. The results of these models were expressed as adjusted odds ratios with 95% confidence interval (CI). Two-tailed Wald test was used to test whether socio-demographic factors were associated with respiratory symptoms at 1% and 5% level of significance. The goodness of fit of each model was tested using Hosmer and Lemeshow statistic [23, 24]. Data management and analysis were performed using STATA 14 (Stata Corp: College Station, TX, USA).

## Results

Descriptive statistics of the respondent’s residential factors, mother’s characteristics, and child characteristics were given in Table 1. About 40% of women exclusively used biomass fuel irrespective of kitchen location; and among the biomass fuel users, approximately 54% had a cooking place within the households. Table 2 demonstrated the prevalence of respiratory symptoms of children which were found higher for in-house biomass fuel users compared with non-biomass fuel users. This rate was 23.0% and 21.9% for in-house and outdoor biomass users respectively, whereas 19.1% for non-biomass users and the association was statistically significant (*p* < 0.01).

**Table 1:** Descriptive statistics of participant’s residential factors, mother’s characteristics, and child characteristics

Exposure variables (*n*)	Percentages
Use of biomass fuel	
Non-biomass user (*n* = 6402)	60.5
In-house biomass user (*n* = 2235)	21.1
Outdoor/separate house biomass user (*n* = 1938)	18.3
Residential factors	
Area of residence	
City corporation: slum (*n* = 4802)	45.4
City corporation: non-slum (*n* = 2433)	23.0
Other urban area (*n* = 3340)	31.5
Migration status	
Non-migrated (*n* = 6484)	61.3
Migrated (*n* = 4091)	38.6
Toilet facility	
Not improved (*n* = 1605)	15.1
Improved (*n* = 8970)	84.8
Sources of water	
Piped water (*n* = 5467)	51.7
Tube well (*n* = 5034)	47.6
Others (*n* = 74)	0.7
Floor materials	
Others (Earth/wood/tiles) (*n* = 3069)	29.0
Concrete/cement (*n* = 7506)	70.9
Garbage disposal	
Not improved (*n* = 5404)	51.1
Improved (*n* = 5171)	48.9
Mother characteristics	
Mothers education	
No formal education (*n* = 1769)	16.7
Primary (*n* = 3213)	30.3
Secondary and above (*n* = 5593)	52.8
Age of the mother (in years)	
Less than 19 (*n* = 964)	9.1
20–29 (*n* = 7037)	66.5
30 and above (*n* = 2574)	24.3
Birth complication of mother	
No (*n* = 7215)	68.2
Yes (*n* = 3360)	31.7
Current working status of the mother	
No (*n* = 8785)	83.0
Yes (*n* = 1790)	16.9
Exposure to media	
No exposure (*n* = 2551)	24.1
At least one media exposed in everyday (*n* = 8024)	75.8
Exposure to NGO	
Not a member of NGO (*n* = 8822)	83.4
At least one NGO member (*n* = 1753)	16.5
Place of hand washing	
Not observed (*n* = 1165)	11.0
Observed (*n* = 9410)	88.9
Child characteristics	
Age of the child (in months)	
0–5 (*n* = 1441)	13.6
6–23 (*n* = 4687)	44.3
24–59 (*n* = 4447)	42.0
Birth order	
1 (*n* = 3639)	34.3
2 (*n* = 3774)	35.6
3+ (*n* = 3162)	29.9
Gender of the children	
Boy (*n* = 5290)	50.0
Girl (*n* = 5285)	49.9
Stunting	
No (*n* = 5887)	55.6
Yes (*n* = 3286)	31.0

**Table 2:** Association between respiratory symptoms of the children and use of biomass fuel, residential factors, and mother and child characteristics

Explanatory variables (*n*)	Respiratory symptoms		
	Absent (8414)	Present (2161)	*p* value
Use of biomass fuel			
Non-biomass user (6402)	80.9	19.1	< 0.001
In-house biomass user (2235)	77.0	23.0	
Outdoor/separate house biomass user (1938)	78.1	21.9	
Residential factors			
Area of residence			
City corporation: slum (4802)	79.4	20.6	0.044
City corporation: non-slum (2433)	81.3	18.7	
Other urban area (3340)	78.6	21.4	
Migration status			
Non-migrant (6484)	80.1	19.9	0.080
Migrant (4091)	78.7	21.3	
Toilet facility			
Not improved (1605)	77.2	22.8	0.011
Improved (8970)	80.0	20.0	
Sources of water			
Piped water (5467)	80.6	19.4	0.020
Tube well (5034)	78.4	21.6	
Others (74)	82.4	17.6	
Floor materials			
Others (Earth/wood/tiles) (3069)	79.2	20.8	0.530
Concrete/cement (7506)	79.7	20.3	
Garbage disposal			
Not improved (5404)	79.1	20.9	0.220
Improved (5171)	80.1	19.9	
Mother characteristics			
Mothers education			
No formal education (1769)	81.9	18.0	0.017
Primary (3213)	78.6	21.3	
Secondary and above (5593)	79.3	20.6	
Age of the mother (in years)			
Less than 20 (964)	77.1	22.9	< 0.001
20–29 (7037)	79.4	20.6	
30 and above (2574)	80.9	19.1	
Birth complication of mother			
No (7215)	82.0	18.0	< 0.001
Yes (3360)	74.3	25.7	
Current working status of the mother			
No (8785)	79.0	21.0	0.002
Yes (1790)	82.4	17.6	
Exposure to media			
No exposure (2551)	78.4	21.6	0.010
At least one media exposed in everyday (8024)	79.9	20.1	
Exposure to NGO			
Not a member of NGO (8822)	80.5	19.5	< 0.001
At least one NGO member (1753)	74.9	25.1	
Place of hand washing			
Not observed (1165)	76.9	23.1	< 0.001
Observed (9410)	79.9	20.1	
Child characteristics			
Age of the child (in months)			
0–5 (1441)	81.8	18.3	< 0.001
6–23 (4687)	77.5	22.6	
24–59 (4447)	81.1	18.9	
Birth order			
1 (3639)	77.1	22.9	< 0.001
2 (3774)	80.7	19.3	
3+ (3162)	81.1	18.9	
Gender of children			
Boy (5290)	78.8	21.3	0.044
Girl (5285)	80.4	19.6	
Stunting			
No (5887)	79.2	20.8	< 0.001
Yes (3286)	75.9	24.1	

The prevalence of respiratory symptoms changed significantly (*p* < 0.05) with the area of residence and the highest was found in other urban areas (21.4%) and the least in city corporation non-slum area (18.7%). Children from households using non-improved toilet facilities (22.8%) and tube well water (21.6%) for their daily use had experienced the highest proportion of respiratory symptoms.

All mother characteristics and child characteristics were significantly associated with the presence of respiratory symptoms of children. Mothers who completed primary (21.3%); and secondary and above (20.6%) education level had children with a higher proportion of respiratory symptoms than mothers having no formal education (18.0%). Children of mothers having any complications at the time of delivery suffered more from respiratory symptoms than children of mothers with no complications (25.7% versus 18.0%). The media played a positive role to reduce the respiratory symptoms of children. Children of mothers exposed to at least one media were less likely to develop respiratory symptoms than children of mothers not exposed to media. Similar results were found for children whose mothers were the member of NGO and having a handwashing place in their household. Children in the age group 6–23 months were at a higher risk than other age groups. The prevalence of respiratory symptoms was found higher (24.1%) in the stunted child.

To find out the unadjusted and adjusted influence of biomass fuel use, four sequential multiple logistic regression models had been fitted and the results shown in Table 3. In the unadjusted model (Model 1), children from households using biomass fuel were significantly (*p* < 0.01) associated with respiratory symptoms. The odds ratio (OR) with 95% CI for in-house and outdoor biomass fuel users were [OR = 1.27; 95% CI: (1.13–1.42)] and [OR = 1.19; 95% CI: (1.05–1.34)] respectively.

**Table 3:** Effects of biomass fuel use, residential factors, and mother and child characteristics on respiratory symptoms based on sequential multiple logistic regression models

Explanatory variables	Model 1	Model 2	Model 3	Model 4
	UOR [95% CI]	AOR [95% CI]	AOR [95% CI]	AOR [95% CI]
Use of biomass fuel				
Non-biomass user	ref	ref	ref	ref
In-house biomass user	1.27 [1.13–1.42]^***^	1.22 [1.07–1.39]^***^	1.17 [1.03–1.34]^**^	1.18 [1.04–1.36]^**^
Outdoor/separate house biomass user	1.19 [1.05–1.34]^***^	1.13 [0.99–1.29]	1.12 [0.97–1.28]	1.13 [0.97–1.30]
Residential factors				
Area of residence				
City corporation: slum		ref	ref	ref
City corporation: non-slum		1.08 [0.96–1.23]	1.09 [0.95–1.25]	1.03 [0.90–1.19]
Other urban area		1.06 [0.92–1.23]	1.04 [0.89–1.20]	0.97 [0.83–1.13]
Toilet facility				
Not improved		ref	ref	ref
Improved		0.84 [0.74–0.96]^***^	0.85 [0.75–0.97]^**^	0.85 [0.74–0.97]^**^
Sources of water				
Piped water		ref	ref	ref
Tube well		1.05 [0.95–1.18]	1.02 [0.92–1.15]	1.02 [0.91–1.15]
Others		0.82 [0.45–1.49]	0.80 [0.43–1.47]	0.70 [0.35–1.40]
Mother characteristics				
Mothers education				
No formal education			ref	ref
Primary			1.22 [1.04–1.41]^***^	1.21 [1.03–1.42]^**^
Secondary and above			1.22 [1.05–1.42]^***^	1.18 [1.00–1.38]^**^
Age of the mother (in years)				
Less than 20			ref	ref
20–29			0.89 [0.76–1.05]	1.05 [0.87–1.26]
30 and above			0.83 [0.69–1.00]	1.00 [0.79–1.25]
Birth complication of mother				
No			ref	ref
Yes			1.54 [1.39–1.70]^***^	1.51 [1.36–1.67]^***^
Current working status of the mother				
No			ref	ref
Yes			0.81 [0.71–0.93]^***^	0.88 [0.76–1.02]
Exposure to media				
No exposure			ref	ref
At least one media exposed in everyday			0.92 [0.81–1.03]	0.90 [0.79–1.02]
Exposure to NGO				
Not a member of NGO			ref	ref
At least one NGO member			1.32 [1.16–1.49]^***^	1.32 [1.16–1.51]^***^
Place of hand washing				
Not observed			ref	ref
Observed			0.86 [0.74–1.00]	0.84 [0.72–0.99]^**^
Child characteristics				
Age of the child (in months)				
0–5				ref
6–23				1.29 [1.10–1.52]^***^
24–59				1.05 [0.89–1.24]
Birth order				
1				ref
2				0.83 [0.73–0.94]^***^
3+				0.80 [0.68–0.94]^***^
Gender of children				
Boy				ref
Girl				0.90 [0.82–1.00]
Stunting				
No				ref
Yes				1.18 [1.06–1.31]^***^
Goodness of fit of the models	Hosmer-Lemeshow chi2 (1)	Hosmer-Lemeshow chi2 (8)	Hosmer-Lemeshow chi2 (8)	Hosmer-Lemeshow chi2 (8)
Test value (*p* value)	0.00 (1.00)	13.84 (0.08)	4.71 (0.78)	7.22 (0.51)

*UOR* unadjusted odds ratio, *AOR* adjusted odds ratio, *CI* confidence interval

^***^p < 0.01

^**^p < 0.05

Adjusted odds ratios were computed by considering all covariates in regression models that were found significantly associated with respiratory symptoms in bivariate analysis. It was observed from Model 2, Model 3, and Model 4 that though adjustment of potential confounders reduced the magnitude of the association between the use of biomass fuel and presence of respiratory symptoms of children under 5 years of age to some extent, the association remained still statistically significant (*p* < 0.01 in Model 1 and Model 2; and *p* < 0.05 in Model 3 and Model 4). After adjusting residential confounders, it was found that mothers who used in-house biomass fuel were 22% [OR = 1.22; 95% CI: (1.07, 1.39)] more likely to have respiratory symptoms of children than non-biomass fuel users. This rate reduced to 17% [OR = 1.17; 95% CI: (1.03, 1.34)] when both residential factors and mother characteristics were adjusted; and to 18% [OR = 1.18; 95% CI: (1.04, 1.36)], while child characteristics were taken into consideration along with confounders considered into the Model 4. The biomass fuel use in an outdoor/separate building for cooking purposes became an insignificant factor for respiratory symptoms when it was adjusted with residential factors.

It was evident from Model 2, Model 3, and Model 4 that improved toilet facility was found to be a significant protective factor of respiratory symptoms. Children under 5 years of age from households having improved toilet facilities were about 15% less likely to have respiratory symptoms compared with children from households not using improved toilet facilities. Observed place of handwashing was found as a protective factor for the development of respiratory symptoms of children under 5 years of age [OR = 0.84; 95% CI: (0.72, 0.99), *p* < 0.05] in Model 4. The respiratory symptoms of children were higher among educated mothers. Odds ratio (OR) of having respiratory symptoms of children, whose mother completed primary, secondary, and above education, compared with non-educated mother was found to be [OR = 1.22; 95% CI: (1.04, 1.41)] and [OR = 1.22; 95% CI: (1.05,1.42)] in Model 3 and [OR = 1.21; 95% CI: (1.03, 1.42)] and [OR = 1.18; 95% CI: (1.00, 1.38)] in Model 4 respectively. If a mother faced any complication during pregnancy, the child was above 50% more likely (*p* < 0.01) to experience respiratory symptoms than a child whose mother did not face such complications. The percentages of respiratory symptom were higher among mothers who are the member of at least one NGO. If a mother was a member of NGO, her child was at least 32% (*p* < 0.01) more likely to have respiratory symptoms compared with a child of non-NGO member mother.

In Model 4, the age of the children was found to be a significant determinant of the presence of respiratory symptoms. Children aged 6 to 23 months were 29% (*p* < 0.01) more likely to have respiratory symptoms compared with other children. Odds ratio of having respiratory symptoms for the second child compared with the first child of parents was found [OR = 0.83; 95% CI: (0.73, 0.94)], whereas it was [OR = 0.80; 95% CI: (0.68, 0.94)] if the birth order of the child is third or above. Malnutrition of children was found to be a significant (*p* < 0.01) risk factors for the presence of respiratory symptoms of children under 5 years of age. The malnourished (stunted) child was 18% more likely to be suffered from respiratory symptoms compared with healthy children.

## Discussion

Using sequential multiple logistic regression modelling to unveil the dynamics of interplay occurring between each of the explanatory variables, this study addressed gaps in the literature regarding using the type of cooking fuel, residential factors, mother and child characteristics, and their relationship with children’s respiratory symptoms in urban areas in Bangladesh.

In this study, we found a high prevalence (23.0%) of respiratory symptoms of under-five children of women who used in-house biomass fuel compared with non-biomass fuel users (19.1%). After adjusting all co-factors in the regression model, it revealed that respiratory symptoms were significantly associated with in-house biomass fuel use. The findings of this study are similar to the results of previous studies conducted in different countries. In Nigeria, the odds of having ARI symptoms among children under 5 years of age were increased (OR = 2.30, CI: 1.26–4.20) among in-house biomass fuel users compared with kerosene/charcoal users at an outdoor or separate place [18]. Households using solid fuel was 1.78 (CI: 1.05–2.99) times more likely to suffer from lower respiratory tract infections (LRTI) than the households using other fuel in India [19]. Children suffering from ARI was 1.79 (CI: 1.02–2.14) times higher among solid fuel users in Nepal [25]. In Bangladesh, nationwide population-based study data revealed that in-house solid fuel use increases the risk of ARI (1.18; CI: 1.08–1.33) symptoms among children under 5 years of age [7]. Another study conducted in the urban slum of Dhaka, Bangladesh, showed that respiratory symptoms like cough, shortness of breath, and chest tightness were significantly high in biomass fuel users than users of fossil fuel [26].

A number of co-factors were explored in this study which showed a significant role in the relationship between biomass fuel uses and respiratory symptoms. The young children aged between 6 and 23 months were 1.29 times more likely to have respiratory symptoms than other children. This is possible because of the practice of keeping these babies with mothers at the time of cooking. This result was supported by the two articles published in Nigeria [18] and Zimbabwe [22]. Vinod Mishra [22] showed that children at age 6–11 months and 12–23 months were 2.24 times and 1.90 times more likely to develop ARI comparing 0–5 months of children respectively. In Nigeria Demographic Health Survey (NDHS) revealed that children of the same age group were 2.66 times and 2.85 times higher chance of developing ARI than 36–59 months of children sequentially [18]. Children who practiced handwashing had less chance of developing a respiratory infection. Almost 66% of children suffered from symptoms of ARI in Nigeria for the poor practice of handwashing [18]. A systematic review was conducted on handwashing and respiratory infection which showed handwashing reduced respiratory infection ranging from 6% to 44% with a pooled reduction of 24% [27]. Faecal contamination on hand is highly associated with gastrointestinal and respiratory symptoms within a household which is possible to control by using improve toilet facilities [28]. More than 50% of children under 5 years of age suffered from respiratory health-related diseases due to the unhygienic sanitation system in Ethiopia [29]. Complication at the time of delivery or pregnancy period plays an important role in child health and is a potential risk factor for respiratory symptoms of a child as well. According to our analysis, the prevalence of mother’s birth complications among in-house biomass fuel users was 34%, whereas these percentages were 31.4% and 30.6% for the non-biomass users and biomass used in a separate room/outdoor. Previous studies have demonstrated the association between preterm birth and chronic respiratory diseases [30]. Preterm infants with bronchopulmonary dysplasia and obstructive lung disease continued to suffer from the disease in adulthood and also were prone to develop the chronic obstructive pulmonary diseases [30]. The pooling of data from 14 European birth cohorts revealed a significant association between preeclampsia and recurrent wheezing among infants up to 2 years of age [31]. Birth order had a positive role in reducing respiratory symptoms. The first child of parents was more vulnerable to this problem and the rate of presence of respiratory symptoms was decreased as birth order increased. This is sometimes possible if the elder child takes care of his/her siblings at the time of the mother’s cooking or working in the kitchen. But this result was opposite to the previous study as they found an increasing prevalence of ARI within higher birth order [18, 22]. Malnutrition is an important risk factor of developing ARI [32] which is also a leading cause of mortality and morbidity among children under 5 years of age [33]. Children of mothers having NGO membership were more vulnerable to respiratory symptoms. This reflects their socioeconomic status because poverty-stricken mothers usually take support from NGOs [34]. Our study findings showed that respiratory symptoms were high among children whose mother’s education level was high. This result is very similar to the findings of a study from Nigeria [18] and a pilot study from Indonesia [35]. The educated mothers are more likely to be employed and usually keep their child alone or in the care of someone whose lifestyle may influence or exacerbate the respiratory symptoms.

In this study, we found that gender has no impact on respiratory symptoms which is similar to the other study findings [18, 22]. Place of residence played a positive role in different studies, but an insignificant association with respiratory symptoms was observed in the final model though it showed significant association in bivariate analysis. It indicates that living in urban slums, non-slum or rest of the urban areas has no influence on the respiratory symptoms of children in Bangladesh.

Four models were used, in this analysis, to adjust the potential confounders. Sequential modelling helps to disentangle the dynamics of interplay occurring between each of the environmental and socio-demographic factors [18]. A similar strategy was also used in articles published in Zimbabwe [22] and Nigeria [18]. This study is not beyond the limitations. For lack of awareness or knowledge on respiratory symptoms, there is a possibility of under-reporting or recall bias among the mothers using biomass during the 2 weeks of this study. Data were collected from self-reported measures on residential factors, mother characteristics, and child characteristics with respiratory symptoms. This may lead to results influenced by endogeneity problems. As this analysis was conducted using data obtained from a cross-sectional study, it is not possible to establish a causal relationship. Though tobacco smoking of parents is a well-known risk factor for respiratory symptoms in children [36], in this study this covariate has not been considered as this information is not available in UHS, 2013. The response variable respiratory symptoms of a child were not validated by a medical examination. Symptoms that are easily recognized by the mother namely cough or fast breathing or shortness of breathing were considered to define the response variable. Seasonality was not considered in this analysis due to the unavailability of data. One prior study found that the mean concentration of dust particles and volatile organic components (VOCs) such as benzene, toluene, and xylene were higher in winter season than summer in urban Dhaka [13]. The trend of emergency department visits at a hospital for asthma also gives the indicator of seasonality. A study conducted in Japan showed that the number of patients visited in the emergency department ranged from 1 to 15 per week and the high number occurred in autumn and spring months, mainly in September, October, and April [37]. The national-level DHS surveys collected data by the month of interview as a proxy seasonal factor and it shows no significant influence on the findings with or without this season factor [38].

## Conclusion

This study found the use of in-house biomass fuel in the kitchen as a significant risk factor associated with respiratory symptoms in children under 5 years of age in urban Bangladesh. Apart from the mother’s educational level and birth complications, other factors like toilet facility, exposure to NGO members, age of the children, birth order, and nutritional status of the child play a significant role in influencing respiratory symptoms. More longitudinal studies should be designed to establish a causal relationship between HAP and respiratory symptoms among children with other possible non-cooking sources of HAP, more direct measures of HAP and clinical procedure.

## Supplementary information

**Additional file 1.** Definition of different co-factor variables used in this article.

## Data Availability

Data for this study are available through the MEASURE Evaluation Data verse website (https://goo.gl/TixL9h).

## Abbreviations

WHO: World Health Organization
UNICEF: United Nations International Children’s Emergency Fund
BUHS: Bangladesh Urban Health Survey
HAP: Household air pollution
LMIC: Low- and middle-income countries
ARTI: Acute respiratory tract infection
ARI: Acute respiratory infection
NGO: Non-government organization
VOCs: Volatile organic compounds
