# Supplementary material for: Association of biomass fuel smoke with respiratory symptoms among children under 5 years of age in urban areas: results from Bangladesh Urban Health Survey, 2013
Source: Environ Health Prev Med. 2019 Nov 27;24:65. doi: 10.1186/s12199-019-0827-3 (PMC6882069; doi:10.1186/s12199-019-0827-3)
Supplement: Supplementary file 1 — Additional file 1. Definition of different co-factor variables used in this article. [file 12199_2019_827_MOESM1_ESM.docx]

**Residence of the respondent:**Residence of the respondent was classified into three groups which are

1. City corporation slum: slum populations in the 9 city corporations in Bangladesh
2. City corporation non-slum: Non-slum populations in the 9 city corporations in Bangladesh
3. Other urban areas: District municipalities and large towns/ Paurashavas with population over 45,000 habitants as listed in the 2011 population census. This domain is referred to as “other urban areas” in urban health survey 2013.

**Toilet facility:** Toilet facility was classified as **improved** (Flush to sewer system/septic tank and ventilated improved pit (VIP)) and **non-improved** (Flush to somewhere else/ DK; Pit latrine without slab /open pit; Bucket toilet; Hanging toilet / hanging latrine; No facility/bush/field).

**Waste disposal:** It is classified as two groups as **improved** which contains waste collected from home/ HH disposes within premises/disposes of in bin outside the house/Burned/ Buried and **non-improved**contains HH disposes of in open spaces outside house/ Other.

**Expose to media:**Data was collected in major three domains of media which are: watching television; newspaper reading and listing radio. If any respondent is exposed to any of the media within a day then it is included in the group as “at least one media expose in every day” otherwise no exposure.

**Place of handwashing:**Interviewers were instructed to observe the place where household members usually washed their hands and also looked for the availability of water supply, had cleansing agents near the place of handwashing. If they found it treated as observed, otherwise non-observed.
